# The impact of the size and angle of the cochlear basal turn on translocation of a pre-curved mid-scala cochlear implant electrode

**DOI:** 10.1038/s41598-023-47133-5

**Published:** 2024-01-10

**Authors:** Irumee Pai, Steve Connor, Charalampos Komninos, Sebastien Ourselin, Christos Bergeles

**Affiliations:** 1 School of Biomedical Engineering and Imaging Sciences, King’s College London, London, UK; 2 St. Thomas’ Hearing Implant Centre, St. Thomas’ Hospital, Guy’s and St. Thomas’ NHS Foundation Trust, 2nd Floor Lambeth Wing, London, SE1 7EH UK; 3 Department of Radiology, Guy’s and St. Thomas’ NHS Foundation Trust, London, UK; 4 Department of Neuroradiology, King’s College Hospital NHS Foundation Trust, London, UK

**Keywords:** Anatomy, Outcomes research, Biomedical engineering, Tomography

## Abstract

Scalar translocation is a severe form of intra-cochlear trauma during cochlear implant (CI) electrode insertion. This study explored the hypothesis that the dimensions of the cochlear basal turn and orientation of its inferior segment relative to surgically relevant anatomical structures influence the scalar translocation rates of a pre-curved CI electrode. In a cohort of 40 patients implanted with the Advanced Bionics Mid-Scala electrode array, the scalar translocation group (40%) had a significantly smaller mean distance A of the cochlear basal turn (*p* < 0.001) and wider horizontal angle between the inferior segment of the cochlear basal turn and the mastoid facial nerve (*p* = 0.040). A logistic regression model incorporating distance A (*p* = 0.003) and horizontal facial nerve angle (*p* = 0.017) explained 44.0–59.9% of the variance in scalar translocation and correctly classified 82.5% of cases. Every 1mm decrease in distance A was associated with a 99.2% increase in odds of translocation [95% confidence interval 80.3%, 100%], whilst every 1-degree increase in the horizontal facial nerve angle was associated with an 18.1% increase in odds of translocation [95% CI 3.0%, 35.5%]. The study findings provide an evidence-based argument for the development of a navigation system for optimal angulation of electrode insertion during CI surgery to reduce intra-cochlear trauma.

## Introduction

Cochlear implants (CIs) are considered to be one of the most successful medical devices. The efficacy and cost effectiveness of CIs for life-changing rehabilitation of disabling hearing loss are well established, with approximately 750,000 implant recipients worldwide^1^. With advances in implant technology and continually expanding clinical indications, the emphasis is increasingly shifting towards improving outcomes and enhancing patient experience.

One of the main areas of interest in CI research is reduction of surgical trauma to the delicate internal structures of the cochlea during electrode insertion. Preservation of low frequency residual hearing may enable electro-acoustic stimulation, whose potential benefit includes improved speech understanding in background noise, music appreciation and sound localization^2–6^. Furthermore, insertion trauma has been linked to the development of intracochlear fibrosis and neo-ossification, which may have a negative impact on speech perception outcomes and revision surgery in the future^7–9^.

A systematic review by Hoskison et al. found an overall 17.6% trauma rate, determined either radiologically or histologically, in adult CI recipients^10^. It has been proposed that CI insertion could be improved with more accurate and consistent electrode insertion, for example in the form of robotic guidance^10,11^. Given that electrode insertion is performed essentially blindly beyond the round window or cochleostomy at present, it would seem a logical initial step to determine the anatomical factors which may be associated with increased insertion trauma. In this context, the most basic surgical aim would be to place the electrode within the scala tympani (ST) compartment of the cochlea without translocation to the scala media (SM) or scala vestibuli (SV), which has been shown to influence CI outcomes^12^. A number of studies to date have examined various cochlear parameters as defined by pre-operative imaging and CI outcomes, the estimated cochlear duct length and angular insertion depth (AID) being the most studied parameters^13–15^. Identification of anatomical factors associated with increased likelihood of scalar translocation could be useful in two broad aspects. Firstly, such knowledge could help inform the electrode choice for individual patients even if the anatomical factor is a “fixed” characteristic that is not possible to manipulate; for example, the clinician may choose or avoid a particular type of electrode array depending on the size of the cochlea. Secondly, it may be possible to identify an anatomical feature that has the potential to be applied to the development of an individualised, imaging-based electrode insertion system.

In this study, the authors explored the hypothesis that scalar translocation of a pre-curved CI electrode can be predicted by 1) the size of the cochlear basal turn and 2) the orientation of the inferior segment of the cochlear basal turn relative to the mastoid segment of the facial nerve, expressed as horizontal and vertical angles in the three-dimensional (3D) space.

## Materials and methods

### Ethical considerations

This retrospective study underwent local institutional review by the Clinical Research Analytics Governance group (CRAG) at Guy’s and St. Thomas’ NHS Foundation Trust and was approved, including waived informed consent (GSTT Electronic Record Research Interface, IRAS ID: 257283, Rec Reference: 20/EM/0112). The study was conducted strictly in accordance with the relevant guidelines and regulations.

### Study cohort

The Auditbase, Picture Archiving and Communication System (PACS) Sectra and Electronic Patient Record databases were searched for all adult and paediatric patients who were implanted with the HiFocus™ Mid-Scala (MS) electrode array (Advance Bionics, Valence, CA, USA) at our institution between March 2013 and July 2018 and had post-operative cone beam computed tomography (CBCT). Exclusion criteria were: congenital cochlear anomalies, acquired pathologies affecting the patency of the cochlear lumen (e.g. labyrinthitis ossificans, otospongiosis, vestibular schwannoma extending into the cochlea) and electrode insertion via cochleostomy. Based on the electrode placement within the scalar chambers as assessed on post-operative CBCT, the study cohort was divided into two groups: those with the electrode entirely within the ST compartment of the cochlea (“ST group”) and those with translocation from ST to SV (“ST-SV group”).

### Angular insertion depth and scala position

**Figure 1:**
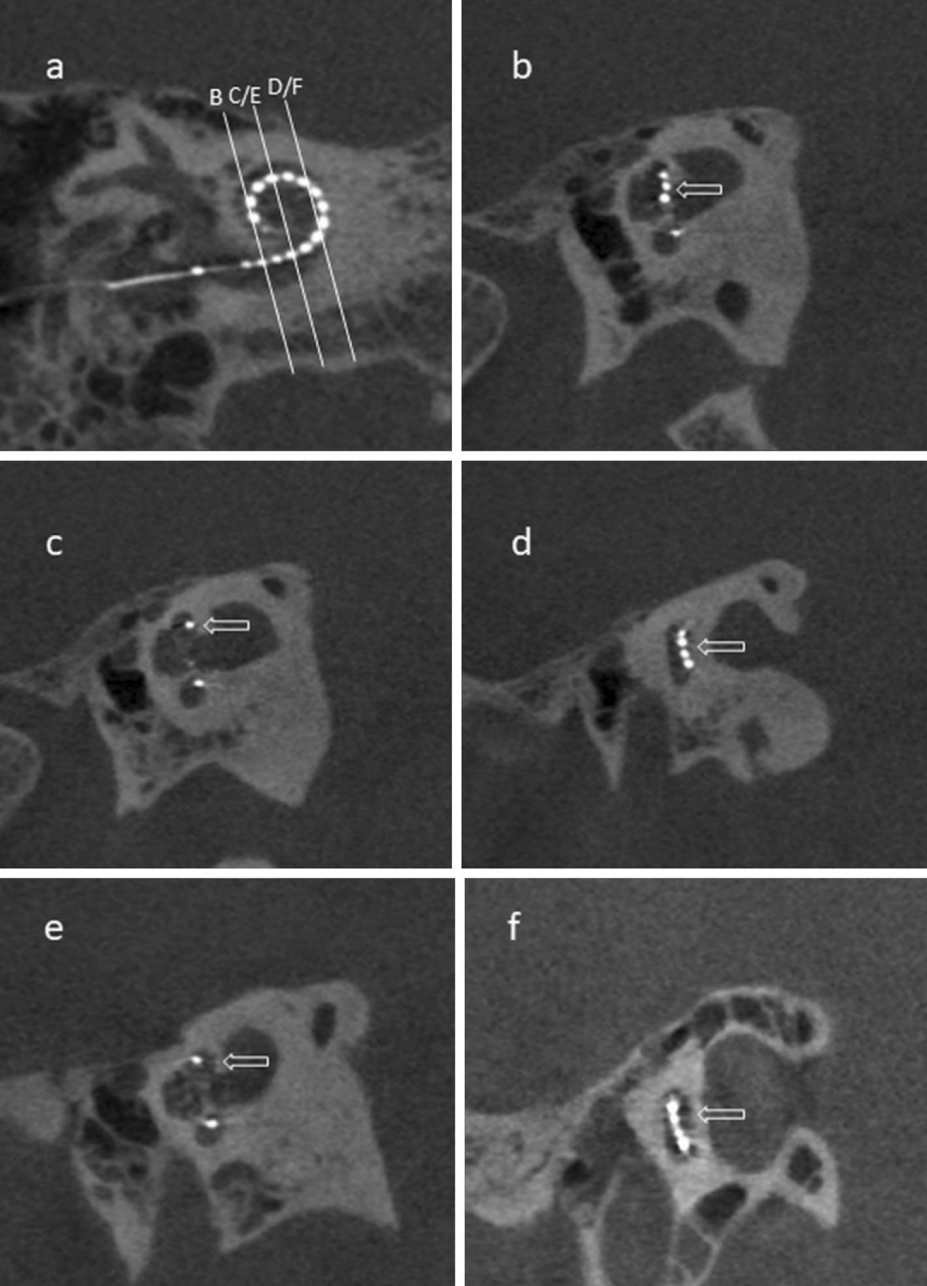
Assessment of scalar translocation. (**a**) Oblique coronal cone beam CT image showing the location of sagittal oblique sections within the descending segment (B), superior and inferior segments (C,E) and ascending segment (D,F). (**b**–**d**) images demonstrate a scala tympani (ST) location of the electrode array throughout without crossing. Arrows in (**b**–**d**) indicate the electrode array in the posterior aspect of the descending turn, superior segment and ascending segments respectively. Note the electrode array is also depicted within the posterior aspect (ST) of the inferior segment in (**c**). (**e**,**f**) images demonstrate crossing of the electrode array from the ST to scala vestibuli (SV) compartments. Arrow in (**e**) indicates the anterior position (SV) of the electrode array whereas the electrode array in the inferior segment is posteriorly positioned (ST). Arrow in (**f**) shows the electrode array passing from posteriorly to anteriorly within the ascending segment.

**Figure 2:**
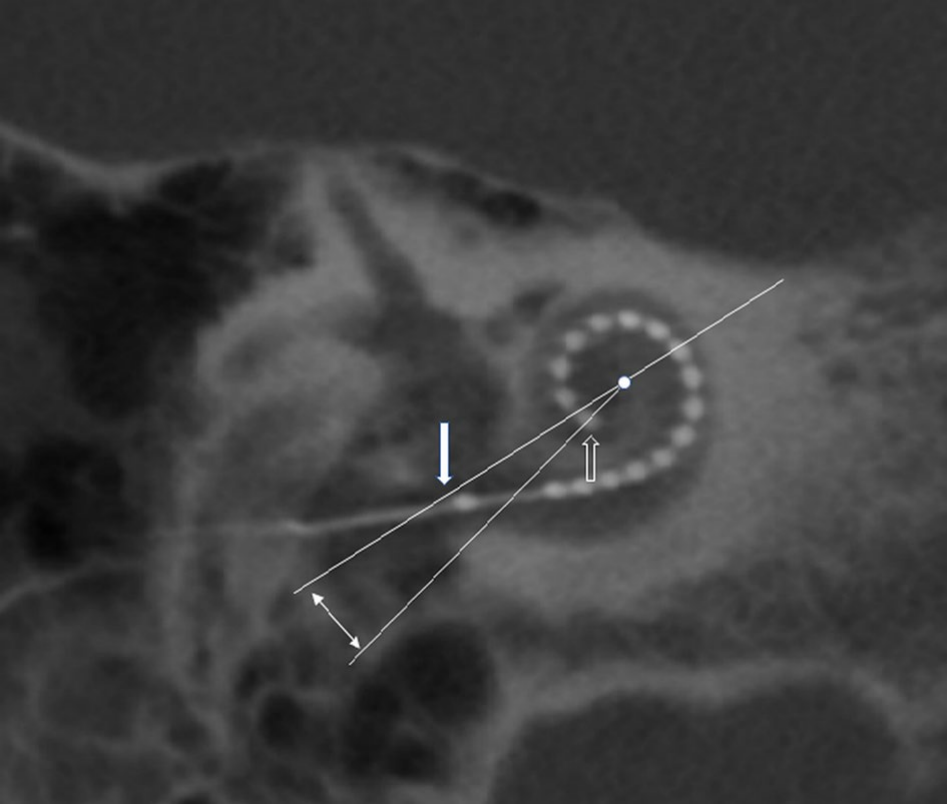
Oblique coronal cone beam CT image illustrating the measurement of angular insertion depth. A 2 mm slab thickness average reconstruction is performed in order to include all electrode contacts within the section thickness. A line is placed in the axis of measurement A from the mid round window (white filled arrow) through the mid-modiolar axis (dot). This bisects the more distal cochlear at the 360° point. An angle (double headed arrow) is then measured between this point and the most distal electrode contact (open arrow). This angle is then added to 360° to determine the angular insertion depth.

CBCT imaging was performed post-operatively using a 3D Accuitomo 170 (J Morita, Kyoto, Japan), model MCT-1, type EX 1/2 F17, with parameters: voltage 80 kV, current 10mA, and 0.125 (0.125 × 0.125 × 0.125). The assessment of scalar translocation and AID was performed by a neuroradiologist (SC) on the Picture Archiving and Communication System (PACS) Sectra software, as per the standard practice at our center^16,17^. The position of the electrode array within the scala chambers was assessed at four locations within the basal turn of the cochlea (mid-inferior segment, ascending segment, mid-superior segment and descending segment), reflecting its antero (ST)—posterior (SV) relationship within the cochlea lumen (Fig. 1a–f). For evaluation of AID, the angle measurement tool of the PACS Sectra software was used on a double oblique coronal reformatted image through the basal turn with a 2mm average slab reconstruction designed to demonstrate the entire electrode array, with the round window as the 0° point. Where the electrode was inserted beyond the basal turn (360°) and into the middle turn of the cochlea, the angle between the round window and the distal electrode contact through the mid-modiolar point was added to 360° (Fig. 2).

### Cochlear dimensions

**Figure 3:**
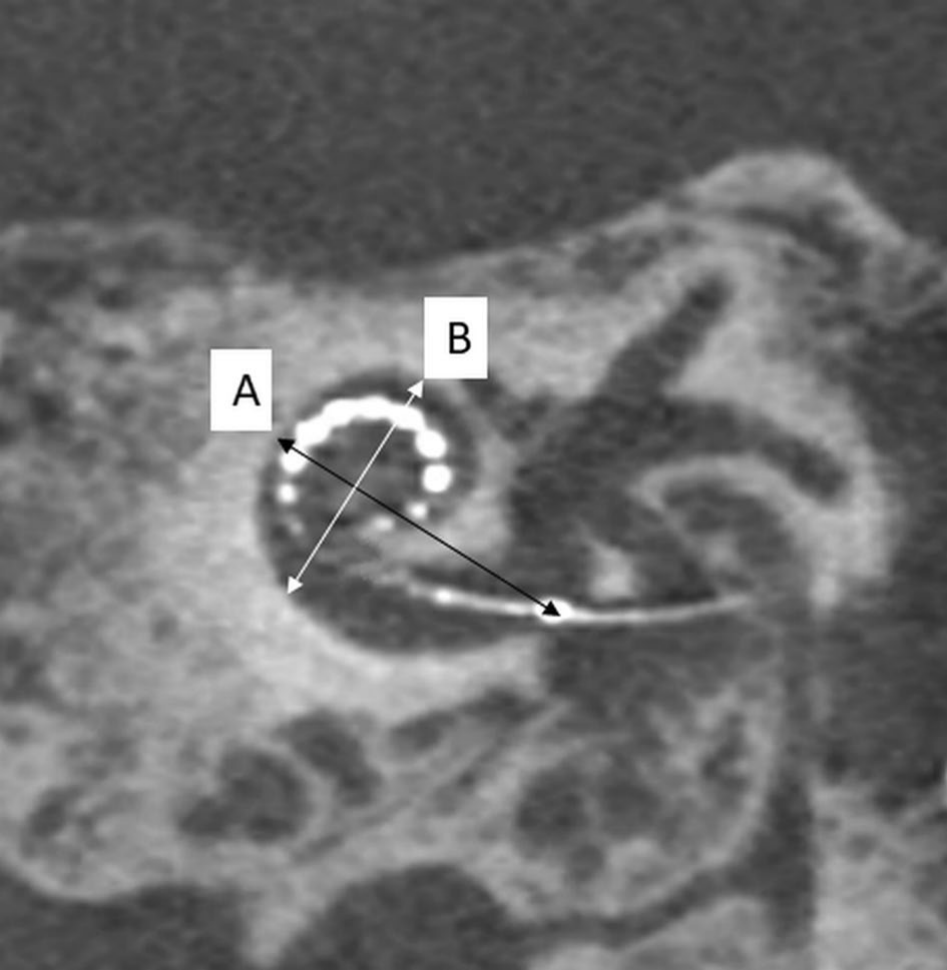
Oblique coronal cone beam CT image demonstrating the distance A and B measurements method. Distance A is indicated by a measurement through the mid-modiolar axis which passes from the round window (at the location of the reference electrode) to the diametrically opposite outer wall of the basal turn of cochlear. Distance B is measured perpendicular to distance A through the mid-modiolar axis, extending between the outer walls of the inferior and superior segments of the basal turn.

The dimensions of the cochlear basal turn were measured in terms of distance A and distance B on a double-oblique paracoronal reformatted image as described by Escude et al.^18^. Images were viewed with a window width/center of 4000/400 and reformatted at 1-mm thick reformat, such that the basal turn from the RW to the opposite outer cochlear wall was visualised on a single image. Distance A was measured as the largest distance from the mid-RW to the opposite wall of the basal turn through the mid-modiolar axis using the measurement tool. Distance B was measured as the distance perpendicular to distance A, joining the outer walls of the superior and inferior segments of the basal turn using the measurement tool and crosshairs as 90° reference (Fig. 3). Both distances were recorded to the nearest 0.1 mm independently by a neuroradiologist (SC) and an implant surgeon (IP).

### Inferior segment of the cochlear basal turn-facial nerve angles

The orientation of the inferior segment of the cochlear basal turn in the 3D space was expressed as horizontal and vertical angles relative to a plane incorporating the mastoid segment of the facial nerve, the most critical structure in defining the facial recess and posterior tympanotomy in the standard transmastoid approach to the RW. These “facial nerve angles” were assessed using the 3D Slicer version 4.11.20210226 (https://www.slicer.org), which is a free, open-source software for visualization, processing, segmentation, registration and analysis of medical images^19–21^.

The inferior segment of the cochlear basal turn was represented by a straight line running through the middle of the RW and the centre of the ST compartment at the mid-inferior segment of the cochlear basal turn, which was achieved by placing fiducials on the above locations in the 3D Slicer. For placement of the mid-ST/mid-inferior segment fiducial, the junction between the inferior and ascending segments was first determined by scrolling through both axial and coronal sections. A fiducial was placed at mid-point between the RW and the junction between the inferior and ascending segments, and then adjusted in the sagittal plane to the middle of the ST compartment. Another fiducial was placed in the centre of the RW, again using all three axial, coronal and sagittal planes. The two fiducials were then joined up with a straight line, hereinafter referred to as the “cochlear line”.

**Figure 4:**
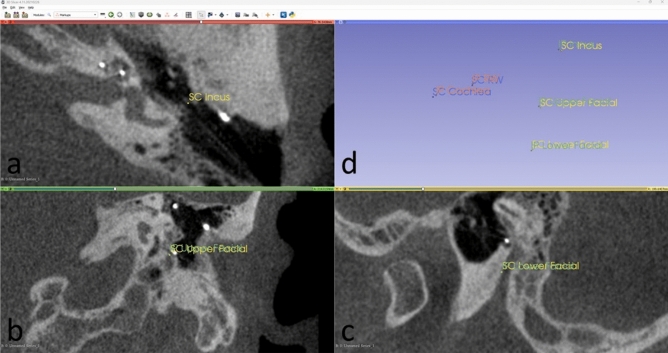
Placements of fiducials in 3D Slicer. Four-up view of fiducial placements by two independent assessors (IP and SC) (**a**) tip of the short process of the incus (axial) (**b**) mastoid segment of the facial nerve at the level of the umbo (coronal) (**c**) mastoid segment of the facial nerve halfway between the upper fiducial and the stylomastoid foramen (**d**) 3D view of all fiducials placed by two assessors.

**Figure 5:**
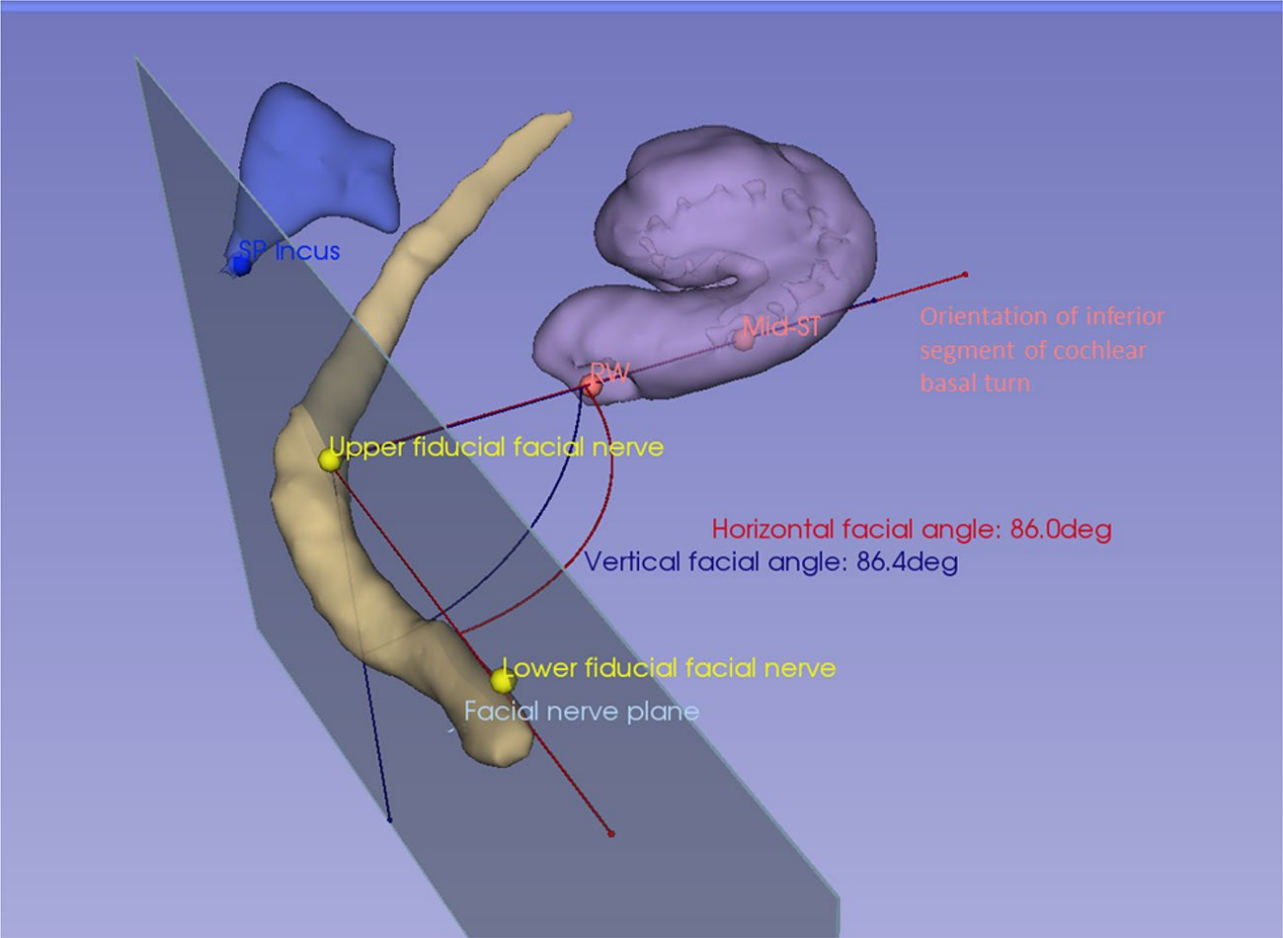
Measurements of horizontal and vertical facial nerve angles in 3D Slicer. 3D volume view generated with 3D Slicer version 4.11.20210226 (https://www.slicer.org), incorporating facial nerve angle measurements and segmentations of the cochlea, facial nerve and incus (3D Slicer). *RW* round window, *SP* short process, *ST* scala tympani.

In order to delineate the mastoid segment of the facial nerve in a manner consistent from case to case, fiducials were placed in the axial, coronal and sagittal planes on the mid-anterior surface of the facial nerve at the level of the umbo of the malleus and a half way between the umbo and the stylomastoid foramen, with a standard obliquity with respect to the long axis of the vestibule (“facial line”). In order to create a plane which incorporated the mastoid segment of the facial nerve and against which the angles of the cochlear line could be measured, a third fiducial was placed on the tip of the short process of the incus since it is one of the surgical landmarks used intraoperatively to determine the location of the second genu of the facial nerve, the starting point of the mastoid segment. A “facial plane” was then created by aligning all three fiducials in the same plane. The fiducials, lines, planes and angles are summarised in Table 1 and illustrated in Figs. 4 and 5. The process of placing the fiducials was performed independently by two observers, a neuroradiologist (SC) and an implant surgeon (IP).

**Table 1:** Fiducial placements for measurement of facial nerve angles.

	Represents	Fiducial 1	Fiducial 2
Cochlear line	Inferior segment of cochlear basal turn	Centre of round window	Centre of scala tympani (“mid-scala”) at mid-inferior segment
Facial line	Mastoid segment of facial nerve	At the level of umbo of malleus	Half way between umbo of malleus and stylomastoid foramen
Short process of incus	Tip of short process of incus	Tip of short process of incus	N/A
Facial plane	Plane incorporating the facial line and short process of incus as determined above, in order to express the orientation of the inferior segment of the cochlear basal turn as angles in the 3D space

For the measurements of the facial nerve angles in 3D Slicer, a Python function (available from the Python Interactor Tool) was developed to read the coordinates of the placed fiducials shown in Fig. 4 and to perform automatic calculation and visualisation of the horizontal and vertical angles as well as the auxiliary lines and planes shown in Fig. 5. In order to facilitate further research and to provide the broader research community with a valuable resource for experimentation and further advancements, our source code has been made available online (https://github.com/RViMLab/SciReports_Cochlear_Angle/tree/main).

### Statistical analysis

All statistical analyses were performed with SPSS v.28.0.1.1 (IBM Cord., Armonk, NY, USA). The inter-rater reliability of the measurements of cochlear dimensions and facial nerve angles was determined by use of the intraclass correlation coefficient (ICC). Since the Shapiro–Wilk test showed the data to be normally distributed, a two-tailed independent Student’s t test was employed to analyse the mean difference between the ST and ST-SV groups for continuous variables. The Chi-square test of independence was used for categorical variables. The threshold for statistical significance was set at alpha = 0.05. In addition, a backward stepwise binary logistic regression was performed to determine whether scalar translocation (the binary dependent variable) could be predicted from distance A, distance B, horizontal facial nerve angle and/or vertical facial nerve angle (the independent variables). At each step, the least correlated variable was removed (exit criterion *p* > 0.10), and the p-value threshold of 0.05 (entry criterion) was used to set a limit on the number of variables used in the final model. Area under the receiver-operating characteristic (ROC) curve was also calculated. Of note, the Bonferroni correction was not applied in view of the fact that the current study had a clear hypothesis, a type II error would be more likely than a type I error, and avoidance of a type I error could be said to be imperative^22,23^.

## Results

### Study population

The search method identified a total of 51 patients who had undergone CI surgery at our institution during the study period and had post-operative CBCT. Of these, 11 cases were excluded from the study for the following reasons: electrode insertion via cochleostomy (n = 5), insufficient clinical data available (n = 4) and indeterminate electrode position with regard to the scalar chambers on CBCT (n = 2). The final analysis therefore included 40 patients that met the inclusion criteria, with a female preponderance (female 25, male 15) and mean age at implantation of 48.8 years (median 48.0, range 4.5–88.0). In all 40 cases, full insertion was achieved intra-operatively and the post-operative CBCT confirmed all 16 electrode contacts to be intra-cochlear. The mean AID was 404° ± 46 (standard deviation, SD) in the ST group (median 410, range 300–450) and 427° ± 46 in the ST-SV group (median 420, range 360–540).

Based on post-operative CBCT, there were 24 patients in the ST group (60%) and 16 patients in the ST-SV group (40%). There was no difference between the two groups in the age at implantation (*p* = 0.43), gender (*p* = 0.74), mean AID (*p* = 0.13) or ear side implanted (*p* = 0.57), with all operating surgeons being right-handed.

### Cochlear dimensions

**Figure 6:**
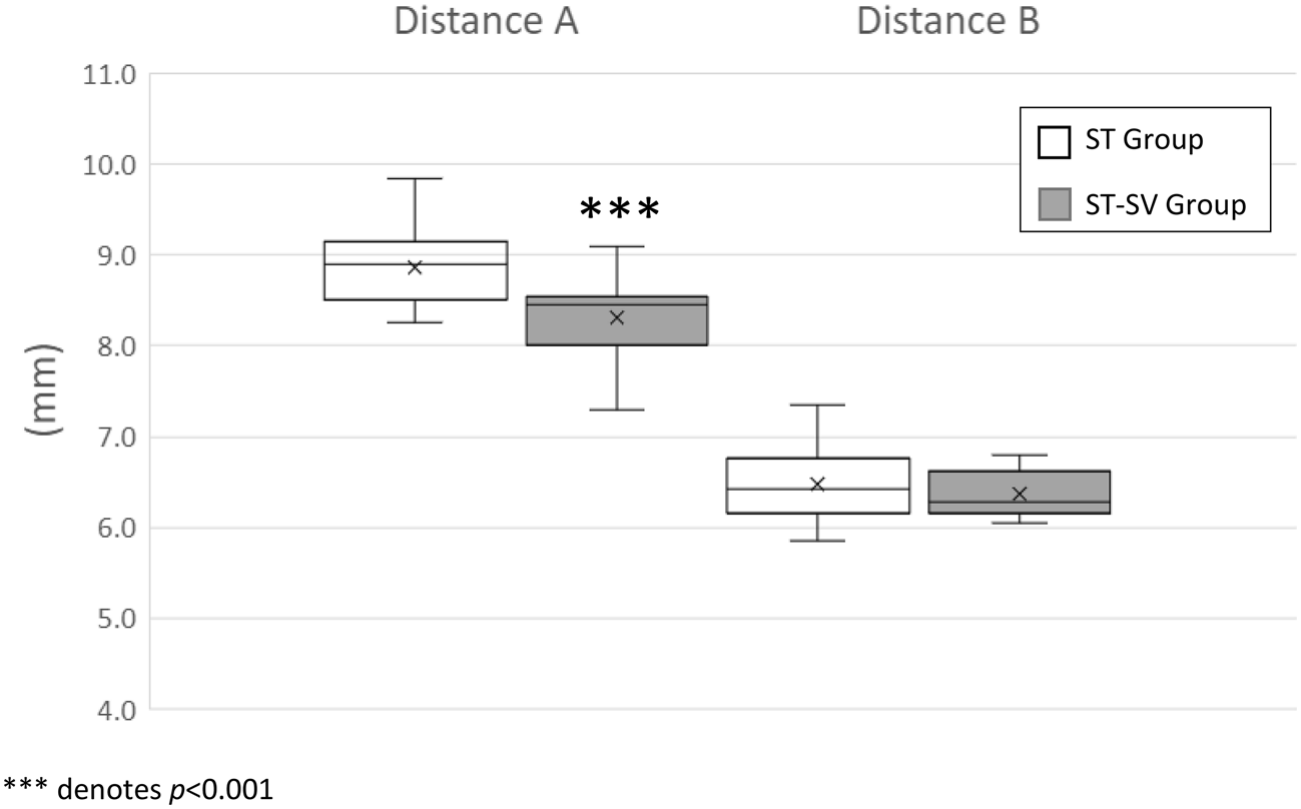
Box and whisker plots for comparison of distance A and distance B between ST and ST-SV groups.

The mean distance A was significantly smaller in the ST-SV group (mean 8.3mm ± 0.4, median 8.5, range 7.3–9.1) than the ST group (mean 8.9 mm ± 0.4, median 8.9, range 8.3–9.9) (*p* < 0.001). There was no statistically significant difference between the two groups in distance B (mean 6.5 mm ± 0.4, median 6.5, range 5.9–7.4 in the ST group; mean 6.4 ± 0.3, median 6.3, range 6.1–6.8 in the ST-SV group) (*p* = 0.36) (Fig. 6). The inter-rater reliability was excellent for both distance A (ICC 0.969, 95% confidence interval (CI) [0.942, 0.984]) and distance B (ICC 0.935 [95% CI 0.868, 0.967]).

### Facial nerve angles

**Figure 7:**
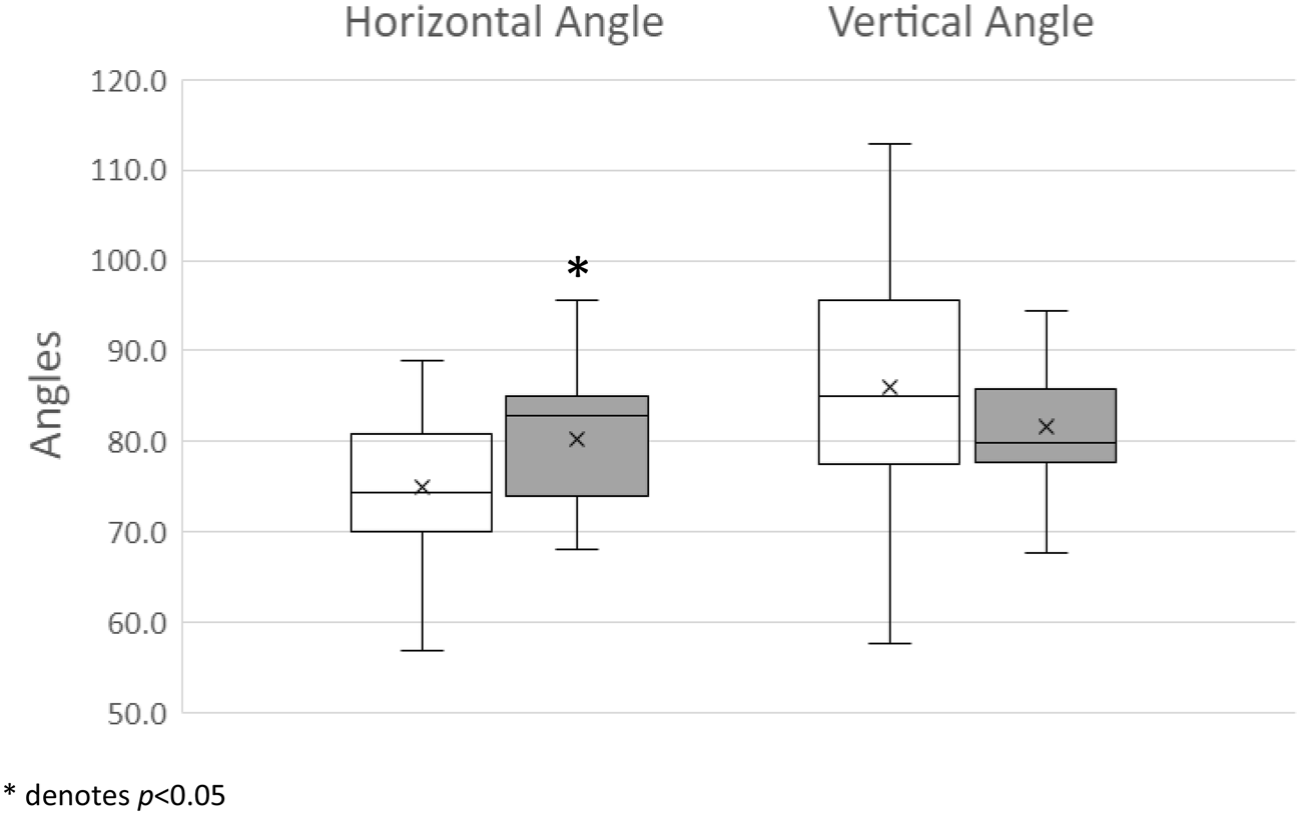
Box and whisker plots for comparison of horizontal and vertical facial nerve angles between ST and ST-SV groups.

The mean horizontal facial nerve angle was significantly wider in the ST-SV group (mean 80.2° ± 7.6, median 82.9, range 68.1–95.7) than in the ST group (mean 74.9° ± 7.7, median 74.3, range 56.9–89.0) (*p* = 0.040). There was no statistically significant difference between the two groups in the vertical facial nerve angle (mean 74.9° ± 7.7, median 74.3, range 56.9–89.0 in the ST group; mean 86.0° ± 16.4, median 85.1, range 57.6–125.1 in the ST-SV group) (*p* = 0.33) (Fig. 7). The inter-rater reliability was excellent for both the horizontal facial nerve angle (ICC 0.986, [95% CI 0.973, 0.992]) and the vertical facial nerve angle (ICC 0.949 [95% CI 0.899, 0.974]).

### Prediction of translocation

**Figure 8:**
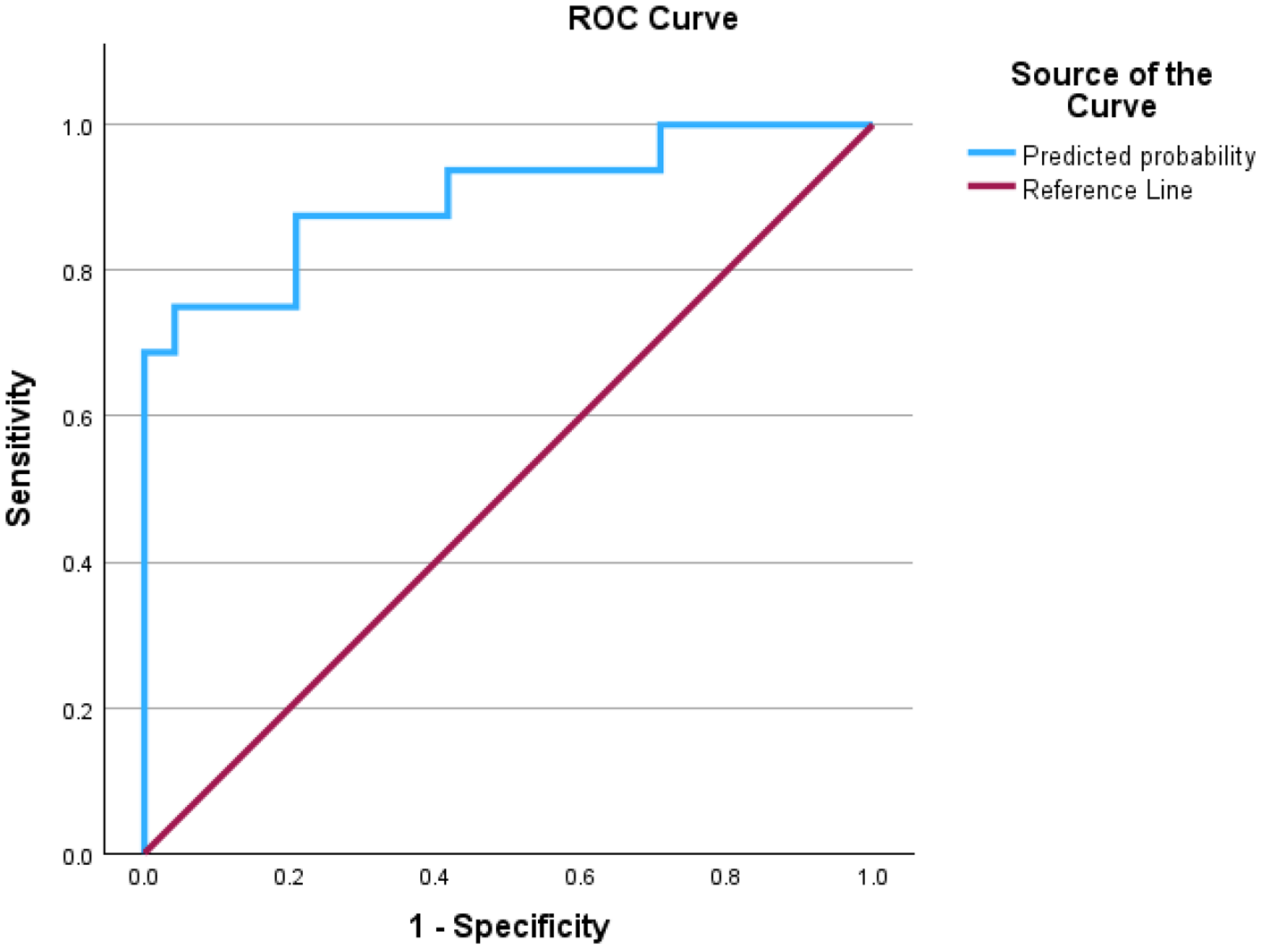
Receiver operating characteristic (ROC) curve of final model.

A backward stepwise binary logistic regression assessed the effect of distance A, distance B, horizontal facial nerve angle and vertical facial nerve angle on the presence of scalar translocation. The Variables in the Equation table is provided in Table 2. The overall model was statistically significant compared to the null model (χ^2^ = 24.89, *p* < 0.001) and the Hosmer–Lemeshow goodness-of-fit test indicated a good logistic regression model fit (χ2 = 6.38, *p* = 0.61). Only distance A (*p* = 0.003) and horizontal facial nerve angle (*p* = 0.017) were statistically significant. The final model with these two independent variables explained between 44.0% (Cox & Snell *R*^2^) and 59.9% (Nagelkerke *R*^2^) of the variance in scalar translocation, indicating a strong relationship, and correctly classified 82.5% of cases. For distance A, every 1mm decrease was associated with a 99.2% increase in odds of translocation [95% CI 80.3%, 100%] whilst for horizontal facial nerve angle, every 1-degree increase was associated with 18.1% increase in odds of translocation [95% CI 3.0%, 35.5%]. The area under the curve (AUC) of the ROC curve for the final model was 0.901 [95% 0.796, 1.006], indicating an outstanding discrimination (Fig. 8).

**Table 2:** Variable in the equation table from backward stepwise logistic regression.

	B	S.E	Wald	df	Sig	Exp(B)	95% CI for EXP(B)	
							Lower	Upper
Step 1^a^								
Distance A	− 5.378	1.758	9.357	1	0.002	0.005	0.000	0.145
Distance B	1.185	1.686	0.494	1	0.482	3.270	0.120	88.974
Horizontal angle	0.218	0.101	4.656	1	0.031	1.243	1.020	1.516
Vertical angle	0.046	0.053	0.766	1	0.381	1.047	0.944	1.161
Constant	17.599	14.595	1.454	1	0.228	43,962,797.751		
Step 2^a^								
Distance A	− 5.048	1.685	8.972	1	0.003	0.006	0.000	0.175
Horizontal angle	0.234	0.102	5.317	1	0.021	1.264	1.036	1.543
Vertical angle	0.053	0.051	1.089	1	0.297	1.055	0.954	1.166
Constant	20.551	13.797	2.219	1	0.136	841,775,701.163		
Step 3^a^								
Distance A	− 4.772	1.605	8.835	1	0.003	0.008	0.000	0.197
Horizontal angle	0.167	0.070	5.695	1	0.017	1.181	1.030	1.355
Constant	27.798	12.272	5.131	1	0.023	1,182,294,718,040		

*B* unstandardized regression weight, *S.E.* standard error, *Wald* Wald test, *df* degrees of freedom, *Sig.* statistical significance of the test, *Exp(B)* odds ratio (predicted change in odds for a unit increase in the predictor).

^a^Variable(s) entered on step 1: distance A, distance B, Horizontal angle, vertical angle.

## Discussion

Electrode placement in the scala tympani (ST) without translocation is the most fundamental prerequisite for preservation of the internal structures of the cochlea during cochlear implant (CI) surgery. In this study, we aimed to investigate the potential anatomical factors that may influence scalar translocation of a pre-curved electrode in normal cochleae via the round window (RW) approach. Our study findings showed that cochleae with scalar translocation of the Advanced Bionics Mid-Scala (MS) electrode array had a significantly smaller distance A (*p* < 0.001) and a wider horizontal angle between the inferior segment of the cochlear basal turn and the mastoid segment of the facial nerve (*p* = 0.04). Moreover, a backward logistic regression model found that both distance A (p = 0.003) and horizontal facial nerve angle (*p* = 0.017) were able to predict scalar translocation with an area under the curve (AUC) value of 0.901 for the final model.

The importance of correct electrode placement within ST is widely recognised both for improved speech perception outcomes and for hearing preservation^12^. The factors that have been found to be associated with higher rates of ST retention include lateral wall arrays^24,25^, electrode insertion via the RW^26^ and prolonged insertion time^27^. A review by Dhanasingh & Jolly from 2019 analysed 1,831 implanted ears reported from 25 peer-reviewed published articles and divided them into pre-curved electrode group (Slim-Modiolar, Contour Advance, Contour, Mid-Scala and Helix) and straight lateral wall group (Slim-Straight, 1J, Standard, Medium, Compressed and FLEX). The authors found the scalar translocation rate to be higher with pre-curved electrodes (32%) than with lateral wall electrode arrays (6.7%) and suggested that lateral wall electrodes be used for all cases without anatomical complications, reserving the use of perimodiolar electrodes for selected clinical situations^25^.

In addition to lower rates of scalar translocation, it has been proposed that lateral wall electrode arrays confer a wider dynamic range, are more likely to achieve preservation of residual hearing at least in the short term, and allow deeper insertion that provides more complete cochlear coverage leading to improved speech perception in noise^28^. However, proponents of peri-modiolar electrodes would argue that there are distinct advantages conferred by having the electrode contacts closer to the neural elements in the modiolus, including narrower spread of excitation, reduced behavioural and electrically-evoked compound action potential thresholds, wider dynamic range and improved speech perception^29–32^. The debate surrounding the choice of CI electrodes is on-going, and the design of an ideal electrode array has yet to be established. Interest therefore still remains in identifying risk factors for scalar translocation with perimodiolar electrodes, and refining the surgical approach to minimise insertion trauma to the cochlea.

The MS electrode, usually classified under peri-modiolar arrays, was designed to reduce the damage to the lateral wall of the cochlea by targeting the largest part of ST and aims to achieve more consistent AID in different size cochleae than electrode arrays that sit closer to the modiolus or in the lateral compartment of ST^33,34^. In our cohort, there was indeed no significant difference in the mean AID between the ST group and ST-SV group despite the mean distance A, representing the size of the cochlear basal turn, being significantly smaller in the ST-SV group. However, the high scalar translocation rate of 40% with the MS electrode array found in this study is also in keeping with previously reported figures which range from 20 to 57% for this electrode type^25^.

Whilst the marked individual variations in the size and morphology of the human cochlea and their potential implications in CI outcomes are well-documented^35–37^, specific anatomic factors that influence scalar translocation have yet to be fully established. In a recent study by Eisenhut et al.^38^, the authors examined scalar translocation in patients implanted with a perimodiolar cochlear implant (CI512, Cochlear Ltd., Sydney NSW, Australia) and reported that it was associated with increased non-linear narrowing of the proximal segment of the cochlear basal turn. It is noted that their study did not find external cochlea diameters including distance A to be significant factors, whereas our findings indicate that scalar translocation of the MS electrode is more likely to occur in cochleae with a smaller distance A and that the current MS electrode design may be better suited to larger cochleae.

In addition to identifying “cochlear factors” that are associated with greater likelihood of scalar translocation, which could aid the clinician to choose the most appropriate electrode array in individual cases, we examined the orientation of the inferior segment of the cochlear basal turn relative to the mastoid segment of the facial nerve as a potential factor that could be used to guide electrode insertion with less trauma. Our study found that the horizontal facial nerve angle varied by 68%, ranging from 56.9° to 95.7°, and the wider this angle, the higher the probability of scalar translocation. This is particularly interesting in view of the findings in a study by Daoudi et al., in which a high rate of translocations occurred with the MS electrode array regardless of whether insertion was manual or with a teleoperated robot^39^. In a subsequent cadaveric study using the same robotic system, Torres et al. were able to reduce insertion trauma and scalar translocation rates of the MS array by adding navigation to robotic insertion in order to align the electrode to the axis of the basal turn of the ST and its subsequent coiling^40^. It therefore seems plausible that, the process of calculating on pre-operative imaging the angulation of the inferior segment of the cochlear basal turn in relation to the plane of the mastoid segment of the facial nerve and the short process of the incus, which are important structures routinely encountered during CI surgery, could be automated and combined with a simple navigation system intra-operatively in order to guide electrode insertion. This is an important consideration, since, unlike other anatomic risk factors that cannot be altered, it is something that is open to surgical manipulation.

In an earlier study by Breinbauer and Praetorius^41^, the authors used the 3D Slicer to segment the cochlea and demonstrated the variability in ideal insertion vectors via cochleostomy and round window approaches in 100 ears, relative to the axial, coronal and sagittal planes. Since these angles are difficult for the operating surgeon to apply to the intraoperative setting, they went on to express the insertion vector as distances in mm to the tip of the short process of the incus and the mastoid segment of the facial nerve. In the current study, we used the same structures to construct a plane against which the orientation of the inferior segment of the cochlear basal turn could be described. Although there were some differences between the two studies in the methodology adopted, both explored the concept of utilising other anatomical structures relevant to and accessible during CI surgery to potentially inform and guide electrode insertion, as part of the effort to reduce intra-cochlear trauma.

Another potential benefit of such an approach would be standardisation of electrode insertion, especially if performed with a mechanical system at a constant and consistent insertion speed. The evidence for factors that influence hearing preservation in CI surgery in the current literature is not conclusive. Various systematic reviews and meta-analyses to date have produced somewhat conflicting outcomes with regard to the effect(s) of the surgical approach (RW insertion vs cochleostomy), electrode insertion speed, choice of electrode type or length, and use of corticosteroids on hearing preservation rates^42–44^. The possibility that such discrepancies may be at least in part due to wide variations in the accuracy of the insertion trajectory cannot be excluded. If the process of electrode placement were to be optimised and made more reliably consistent as far as possible through mechanical insertion at a constant speed along a pre-determined ideal insertion trajectory, it would reduce the impact of a potentially significant variable.

The authors acknowledge that this study has a number of limitations. Firstly, only one specific type of an electrode array was evaluated and the findings cannot be generalised to other types of electrodes. Our study did not include the other type of peri-modiolar electrodes, so-called “modiolus-hugging”, as they are rarely used in our centre. As for the lateral wall electrodes which have much lower scalar translocation rates as already discussed, a similar study on anatomic parameters will require a different outcome measure, for example hearing preservation rates. Secondly, the facial nerve angles measured in this study are not transferable to the intra-operative setting in the current formats, as the mastoid segment of the facial nerve is not routinely delineated along its entire length during CI surgery. Nonetheless, this does not detract from the main study findings, as the anatomical structures used in this study to define the relative angulation of the inferior segment of the cochlear basal turn are pertinent to CI surgery in terms of the access to the cochlea. Finally, the relative importance of the diameter of the cochlear basal turn and the horizontal facial nerve angle is not clear; in other words, it cannot be determined from the study findings whether aligning the insertion trajectory to the orientation of the inferior segment of the cochlear basal turn would necessarily reduce the scalar translocation rate in smaller cochleae. That said, the authors believe that the current study has successfully identified two important anatomic factors that are associated with insertion trauma and produced the evidence base to support the development of imaging-based navigation in CI surgery to optimise the insertion trajectory.

## Conclusion

The probability of scalar translocation of the pre-curved MS electrode array increases as distance A of the cochlear basal turn decreases and the angle between the inferior segment of the cochlear basal turn and the mastoid segment of the facial nerve increases.

## Data Availability

The data from the current study are available from the corresponding author on reasonable request.
