# Publisher Correction: The impact of the size and angle of the cochlear basal turn on translocation of a pre‑curved mid‑scala cochlear implant electrode

**DOI:** 10.1038/s41598-024-57146-3

**Published:** 2024-03-19

**Authors:** Irumee Pai, Steve Connor, Charalampos Komninos, Sebastien Ourselin, Christos Bergeles

**Affiliations:** 1 School of Biomedical Engineering and Imaging Sciences, King’s College London, London, UK; 2 St. Thomas’ Hearing Implant Centre, St. Thomas’ Hospital, Guy’s and St. Thomas’ NHS Foundation Trust, 2nd Floor Lambeth Wing, London, SE1 7EH UK; 3 Department of Radiology, Guy’s and St. Thomas’ NHS Foundation Trust, London, UK; 4 Department of Neuroradiology, King’s College Hospital NHS Foundation Trust, London, UK

Correction to: *Scientific Reports* 10.1038/s41598-023-47133-5, published online 10 January 2024

The Acknowledgements section in the original version of this Article was omitted. The Acknowledgements section now reads:

“This work was supported by core funding from the Wellcome/EPSRC Centre for Medical Engineering [WT203148/Z/16/Z]. For the purpose of Open Access, the Author has applied a CC BY public copyright license to any Author Accepted Manuscript version arising from this submission.”

The original Article has been corrected.

